# Bitter Taste Receptor 46 (hTAS2R46) Protects Monocytes/Macrophages from Oxidative Stress

**DOI:** 10.3390/ijms25137325

**Published:** 2024-07-03

**Authors:** Maria Talmon, Lara Camillo, Ilaria Vietti, Federica Pollastro, Luigia Grazia Fresu

**Affiliations:** 1 Department of Pharmaceutical Sciences, University of Piemonte Orientale, 28100 Novara, Italy; federica.pollastro@uniupo.it; 2 Department of Health Sciences, School of Medicine, University of Piemonte Orientale, 28100 Novara, Italy; lara.camillo@med.uniupo.it (L.C.); 20030964@studenti.uniupo.it (I.V.)

**Keywords:** monocytes, macrophages, bitter taste receptors, absinthin, oxidative stress

## Abstract

Bitter taste receptors (TAS2Rs) are not only responsible for taste perception in the oral cavity, but are spread throughout the body, generating a widespread chemosensory system. In humans, 25 subtypes have been identified and are differentially expressed in tissues and organs, including in the immune system. In fact, several TAS2R subtypes have been detected in neutrophils, lymphocytes, B and T cells, NK cells, and monocytes/macrophages, in which they regulate various protective functions of the innate immune system. Given its recognized anti-inflammatory and antioxidant activity, and the generally protective role of bitter taste receptors, in this work, we studied TAS2R46’s potential in the protection of human monocyte/macrophage DNA from stress-induced damage. Through both direct and indirect assays and a single-cell gel electrophoresis assay, we demonstrated that absinthin, a specific TAS2R46 agonist, counteracts the release of reactive oxygen species (ROS) and reactive nitrogen species (RNS) and reduces DNA damage in both cell types. Even though the release of ROS from monocytes/macrophages is fundamental for contrast pathogen agents, supraphysiological ROS production impairs their function, finally leading to cell death. Our results highlight TAS2R46 as a novel player involved in the protection of monocytes and macrophages from oxidative stress damage, while simultaneously supporting their antimicrobial activity.

## 1. Introduction

Bitter taste receptors (TAS2Rs) in the oral cavity have always been considered a protective weapon against potentially poisonous or toxic bitter foods and drinks, but in the last twenty years, their protective roles in other organs have also been described [1]. They seem to play a role in surveillance for harmful molecules or pathogens, with different mechanisms depending on the anatomical localization and physiology of the specific organ, such as through their high anti-inflammatory and antioxidant potential. Indeed, several studies have revealed the expression of TAS2Rs in the heart, in the gastrointestinal tract, in the pulmonary system, in the brain, and, importantly for our study, in immune cells [2], and they are thought to generate a whole chemosensory network. For example, in cardiomyocytes and fibroblasts of the heart, they are able to sense and monitor inflammatory and oxidative stress [3,4]. Meanwhile, in the gut epithelium, bitter ligands play a role in both hormonal secretion [5,6] and protective immunity [7], demonstrating that they are involved in inflammatory gut diseases. Numerous investigations have been carried out in the pulmonary system, in which bitter receptors have been shown to play a complex protective role through signalling cascades [2,8]. In particular, anti-inflammatory and antioxidant potential at the level of epithelial cells has been demonstrated [9]; for example, the receptors have bronchodilator efficacy in the smooth pulmonary muscle [10,11], which protects the lungs from pathogens and harmful substances. The involvement of TAS2Rs in the brain is more controversial, as in physiological conditions, they seem to prevent neuro-inflammation phenomena (confirming their protective role), but in conditions involving particular changes to the signalling cascade, they represent a trigger for neuro-inflammation and support its progression [12,13]. In all situations, however, the inflammatory state provides for the induction of oxidative stress, sustained by a massive cellular release of free oxygen and/or nitrogen radicals [14], with the fundamental involvement of the innate immune system, in which monocytes and macrophages are the most representative players [15]. These cells not only orchestrate the inflammatory process by releasing pro-inflammatory mediators, but also support the resolution of inflammation by triggering anti-inflammatory pathways [16] due to their ability to acquire different phenotypes depending on the external milieu [17,18]. The expression of TAS2R subtype 38 in human monocytes and in the myeloid cellular line was discovered by Maurer et al. in 2015 [19], and later, different subtypes (including TAS2R46) and their functions were demonstrated in other circulating immune cells [20,21]. Monocytes are precursors for nonclassical monocytes depending on the milieu conditions [22,23,24] and have also been shown to differentiate either versus macrophages or dendritic cells (DCs) after migrating to tissue [16,25]. While macrophages have been shown to express several TAS2R subtypes [19,20], so far, these receptors have not been characterized in DCs [26], although Yasutomi et al. demonstrated an anti-inflammatory effect of erythromycin on dendritic cells that was probably TAS2R-mediated thanks to its lactone structure [27]. Our previous study [9] focused on the effect of absinthin on human bronchoepithelial cells, and we demonstrated that this bitter agonist exerts anti-inflammatory and antioxidant activity through TAS2R46, inhibiting the PMA-induced production of the superoxide anion.

In this study, we focused our attention on the expression and function of subtype TAS2R46 in human monocytes/macrophages under an oxidative burst challenge to deepen our understanding of its antioxidative potential and DNA-protective role. We found that the specific bitter agonist absinthin protects DNA following oxidative-stress-induced damage in monocytes and macrophages.

## 2. Results

### 2.1. hTAS2R46 Is Expressed Differently in Monocytes and Macrophages

**Figure 1:**
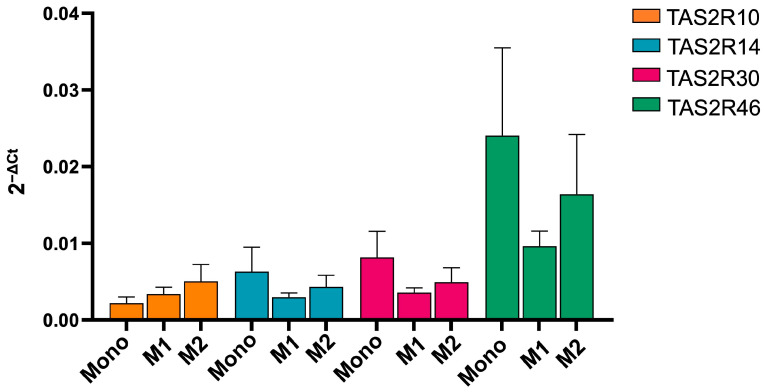
Gene expression of TAS2R in monocytes (Mono) and M1 and M2 macrophages. Data are mean ± SEM of 3 independent experiments and expressed as 2^−ΔCt^.

We first quantified the expression of the TAS2R receptor subtypes towards which absinthin has affinity, such as TAS2R10/14/30 and 46, in monocytes and in M1 and M2 macrophages using qPCR. Figure 1 clearly shows that TAS2R46 is the most expressed subtype.

**Figure 2:**
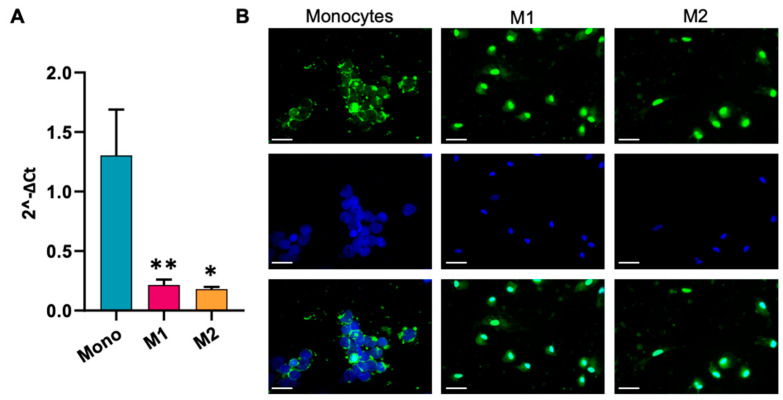
Expression of TAS2R46 in human monocytes and monocyte-derived macrophages. (**A**) qPCR analysis of TAS2R46 in monocytes and M1 and M2 macrophages. Data are mean ± SEM of four independent experiments. ** *p* < 0.01; * *p* < 0.05 vs. Mono (monocytes). (**B**) Representative images of immunofluorescence analysis of hTAS2R46 expression on monocytes (Mono) and M1 and M2 macrophages. Green: hTAS2R46; blue: DAPI (nuclei). Scale bar: 25 µm.

We therefore focused our attention on TAS2R46, whose expression was evaluated using a more sensitive approach (TaqMan assay) for mRNA levels (Figure 2A) and using immunofluorescence for protein expression (Figure 2B). Figure 2A shows that the mRNA expression of the receptor in monocytes was significantly higher than that in the different macrophage populations. We also analysed the protein expression of the receptor using an immunofluorescence assay, confirming that monocytes have higher expression of TAS2R46 compared to macrophages (Figure 2B).

### 2.2. Absinthin Modulates the Cell Phenotype

**Figure 3:**
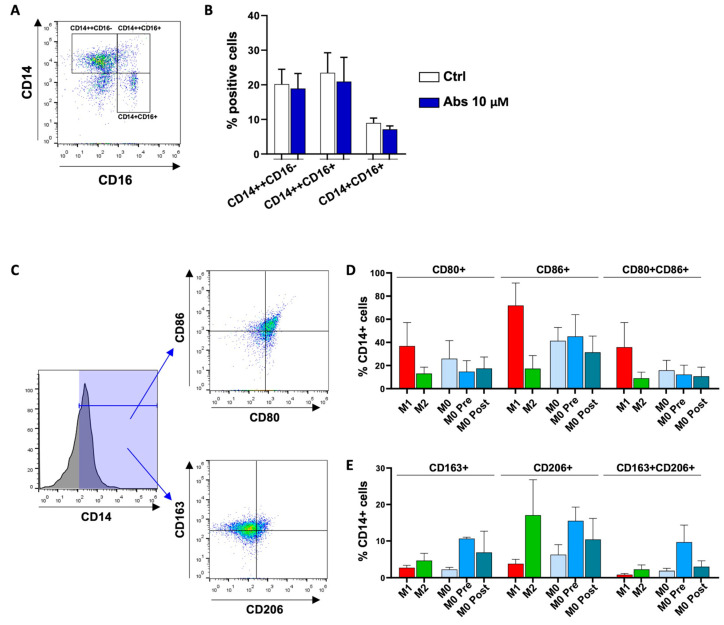
Immunomodulatory effect of absinthin on monocytes and macrophages. (**A**) Gating strategy for monocyte phenotype analysis (dot plot). (**B**) Monocytes were treated with 10 μM absinthin for 1 h and then stained with APC anti-CD14 and FITC anti-CD16 antibodies. (**C**) Gating strategy for macrophage phenotype analysis. CD14+ cells were selected (histogram), and then, we evaluated the positivity to other CD markers (dot plots). (**D**,**E**) M0 were obtained from monocytes stimulated with absinthin (10 μM) and then left to differentiate in enriched medium with 20% FBS (M0 pre) or obtained from monocytes left to differentiate in enriched medium with 20% FBS. Then, fully differentiated M0 were stimulated with 10 μM absinthin (M0 post). Cells were stained with the following antibodies: FITC anti-CD80 and PE anti-CD86 for the M1 phenotype (**D**) and PerCP anti-CD206 and FITC anti-CD163 for the M2 phenotype (**E**). Results are expressed as the percentage of positive cells for each marker of the total of the CD14+ cells. M1 and M2 represent the positive control for macrophage marker expression.

Through the expression of surface markers, we were able to evaluate the effect of absinthin in driving both the polarization of monocytes (CD14 and CD16) and their differentiation into macrophages (CD80 and CD86 for the M1 phenotype, and CD206 and CD163 for the M2 phenotype). Figure 3A shows the gating strategy for monocytes, while in Figure 3B, we demonstrate that the percentages of classical (CD14++/CD16−), non-classical (CD14+/CD16+), and intermediate (CD14++/CD16+) monocyte populations were similar in treated and untreated cells; therefore, absinthin did not induce monocyte polarization.

We thought it might be interesting to evaluate the ability of absinthin to drive monocytes’ differentiation toward macrophages (M0 pre) or to shift the M0 macrophages towards the anti- or pro-inflammatory phenotype (M0 post). M0 macrophages represent an “artificial” population of resting macrophages, waiting to be committed towards a pro- or anti-inflammatory phenotype [28]. In fact, they express surface markers typical for both M1 and M2 phenotypes but at intermediate levels compared to their respective fully differentiated controls (Figure 3D,E). They are therefore a good model with which to evaluate the immunomodulatory potential of a molecule. As shown in Figure 3D, the expression of the pro-inflammatory M1 phenotype marker appeared to be negatively modulated by absinthin, particularly CD80, especially in M0 pre-macrophages (Figure 3D). The expressions of CD163 and CD206 (M2 phenotype markers) were simultaneously enhanced in the same cell population (Figure 3E). Hence, these results demonstrated that absinthin was able to drive macrophage differentiation toward an anti-inflammatory phenotype.

### 2.3. Absinthin Reduced the PMA-Induced Oxidative Burst in Monocytes and Macrophages

Monocytes and macrophages are major producers of free radicals after stimulation [15]. In fact, immune cells rapidly release ROS as an immunological defence in the presence of different pathogens, and it is possible to mimic this in vitro by using chemical triggers for oxidative burst, such as phorbol 12-myristate 13-acetate (PMA) [23]. We therefore stimulated cells with PMA in order to evaluate the antioxidant ability of absinthin. We performed two different experimental tests: an indirect analysis to evaluate the nmoles of reduced cytochrome C using spectrophotometrical analysis, and a direct approach that evaluated the % of ROS/RNS-producing cells through a fluorimetric assay. Moreover, we evaluated the gene expression of the antioxidant enzymes superoxide dismutase (SOD) 1 and glutathione peroxidase 1 (GPX1).

#### 2.3.1. Effect of Absinthin on Monocytes

**Figure 4:**
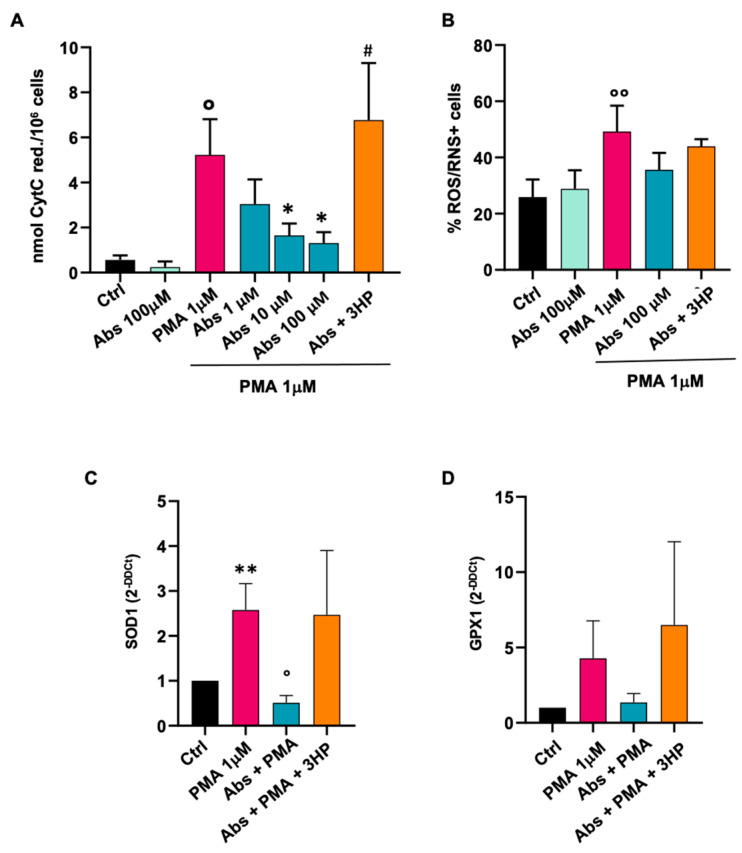
Effect of absinthin on oxidative stress in human monocytes. Cells were pre-incubated with absinthin (Abs) at the indicated concentration with/without the hTAS2R46 antagonist 3HP (10 μM) and then stimulated with PMA (1 μM) for 30 min. (**A**) Dose-dependent decrease in the PMA-oxidative burst in monocytes pre-incubated with absinthin for 1 h and then stimulated with indicated drugs. Data are expressed as nmol of reduced cytochrome C/10^6^ cells and are the mean ± SEM of 6 independent experiments, analysed using one-way ANOVA with a Kruskal–Wallis test for multiple comparisons. Significance levels: ° *p* < 0.05 and °° *p* < 0.01 vs. Ctrl; * *p* < 0.05 vs. PMA; # *p* < 0.05 vs. Abs 10 μM. (**B**) Cytofluorimetric analysis of ROS/RNS-positive cells pre-incubated with absinthin for 1 h, in the presence or absence of the indicated drugs. Data are mean ± SEM of 4 independent experiments. (**C**) qPCR analysis of SOD1 expression after monocyte pre-incubation with absinthin for 6 h and then stimulation with indicated drugs. Data are expressed as mean ± SEM of 4 independent experiments. (**D**) qPCR analysis of GPX1 expression after monocytes were pre-incubated with absinthin for 6 h and then stimulated with indicated drugs. Data are expressed as mean ± SEM of 4 independent experiments Significance levels: ** *p* < 0.01 vs. Ctrl; ° *p* < 0.05 vs. PMA. Abs, absinthin; Ctrl, control (unstimulated cells); PMA, phorbol 12-mystrate 13-acetate; 3HP, 3-hydroxypelenolide.

As reported in Figure 4A, the basal amount of O_2_^-^ in unstimulated monocytes was not affected by absinthin alone at the highest concentration used, while 1 μM PMA administered for 30 min triggered an increase to about six times the basal level. The pretreatment of cells with absinthin significantly counteracted PMA’s effect in a dose-dependent manner, and this could be reverted by the coadministration of the TAS2R46 antagonist 3-hydroxypelenolide (3HP; Figure 4A), demonstrating bona fide hTAS2R46-dependent results.

We then evaluated the monocytes’ responsiveness to PMA through a FACS analysis (Figure 4B), evaluating the percentage of cells found to be positive with the ROS/RNS probe. As shown in Figure 4B, the results showed the same trend as the previous ones; that is, the percentage of positive cells increased after challenge with PMA, and this could be reverted by absinthin.

It was therefore interesting to evaluate the change in the expression of two of the antioxidants most involved in the protection of cells from oxidative stress (that is, SOD1 and GPX1 [29,30] after PMA-oxidative stress) and the possible modulation by absinthin. As shown in Figure 4C (SOD1) and Figure 4D (GPX1), absinthin affected the antioxidative defences in monocytes by changing the gene expression of both enzymes. In fact, PMA induced a significant increase in both SOD1 and GPX1, an indication of oxidative burst induction. However, in the presence of absinthin, the oxidative defence pathway was not induced due to a reduction in the production of reactive oxygen species; therefore, absinthin inactivated the antioxidant defences of the cell. The bitter antagonist 3HP reverted the effect of absinthin.

#### 2.3.2. Effect of Absinthin on M1 and M2 Macrophages

**Figure 5:**
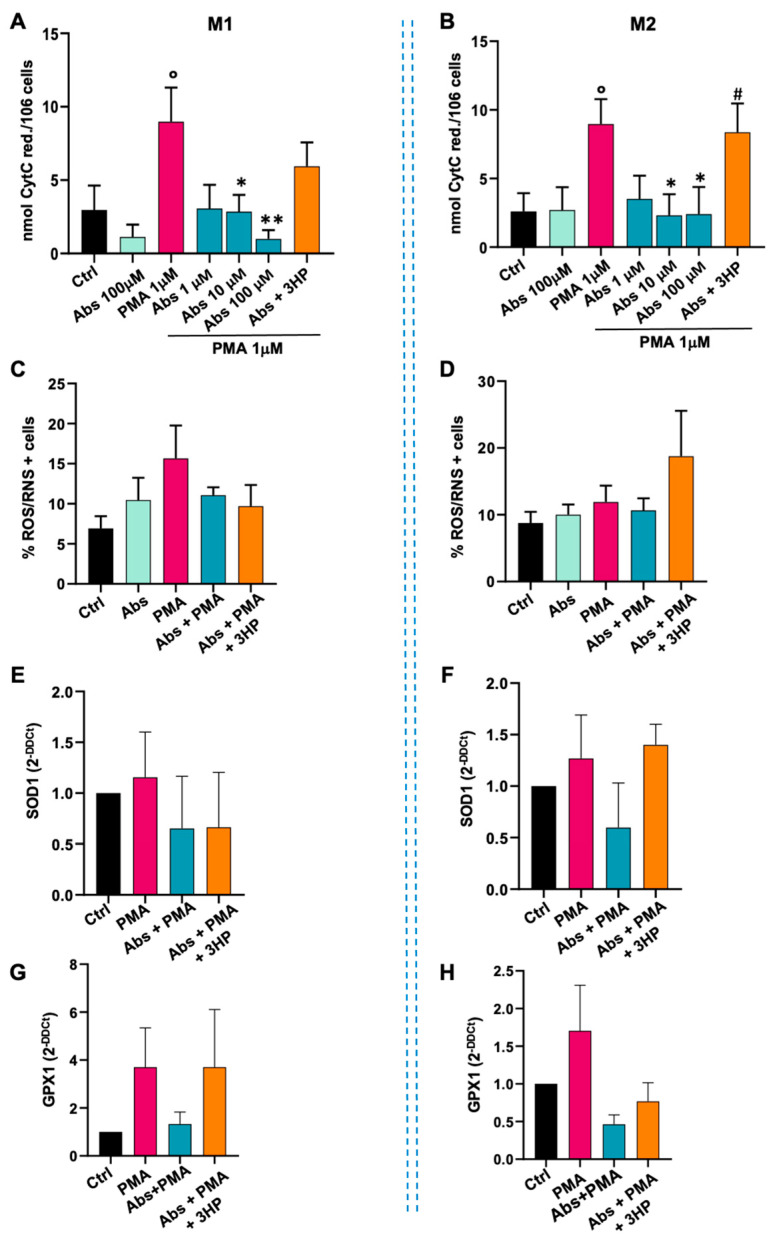
Effects of absinthin on oxidative stress in M1 and M2 macrophages. Fully differentiated macrophages were incubated with absinthin at the indicated concentrations with/without the hTAS2R46 antagonist 3HP (10 μM) and then stimulated with PMA (1 μM) for 30 min. (**A**) PMA-induced oxidative burst in M1 macrophages and (**B**) M2 macrophages. Data are expressed as nmol of reduced cytochrome C/106 cells and as the mean ± SEM of 6 independent experiments. (**C**) Cytofluorimetric analysis of ROS/RNS-positive M1 macrophages and (**D**) positive M2 macrophages, in the presence or absence of the indicated drugs. Data are expressed as the mean ± SEM of 4 independent experiments. (**E**) qPCR analysis of SOD1 expression in M1 macrophages and (**F**) M2 macrophages. (**G**) qPCR analysis of GPX1 expression in M1 macrophages and (**H**) M2 macrophages. Data are expressed as mean ± SEM of 4 independent experiments. Results were analysed using one-way ANOVA with a Kruskal–Wallis test for multiple comparisons. Significance levels: ° *p* < 0.05 vs. Ctrl; * *p* < 0.05 and ** *p* < 0.01 vs. PMA; # *p* < 0.05 vs. Abs 10 μM. Abs, absinthin; Ctrl, control (unstimulated cells); PMA, phorbol 12-mystrate 13-acetate; 3HP, 3-hydroxypelenolide.

The untreated monocytes were subsequently differentiated into M1 and M2 macrophages and then stimulated with absinthin and PMA to evaluate the ability of the bitter ligand to modulate the oxidative pathway in fully differentiated cells. As shown in Figure 4, absinthin at the highest concentration tested failed to induce an increase in superoxide anion in either M1 (Figure 5A) or M2 (Figure 5B) macrophages, while PMA more than doubled the release compared to basal levels in both macrophage populations. As in monocytes, absinthin significantly reduced the PMA-induced oxidative burst in a dose-dependent manner in both M1 (Figure 5A) and M2 (Figure 5B) macrophages compared to control cells. The inhibition of absinthin with the bitter antagonist 3HP reverted the effect, confirming that these results were due to TAS2R46 activity. We subsequently evaluated ROS/RNS production and, as seen in monocytes, we recorded an increase in the percentage of cells positive for the probe after PMA treatment in M1 (Figure 5C) that was reduced by absinthin. Interestingly, in M2 macrophages, the treatments did not produce variations in ROS production, probably because these cells produce fewer nitrogen and oxygen radicals (Figure 5D).

To establish the first general insights into how absinthin may be able to modulate the oxidative burst process, we analysed specific enzymes involved in ROS protection. As shown in Figure 5, absinthin reverted the PMA-induced overexpression of SOD1 (Figure 5E,F) and of GPX1 (Figure 5G,H) to the control levels in both macrophage populations.

#### 2.3.3. Effect of Absinthin on Monocyte Differentiation

**Figure 6:**
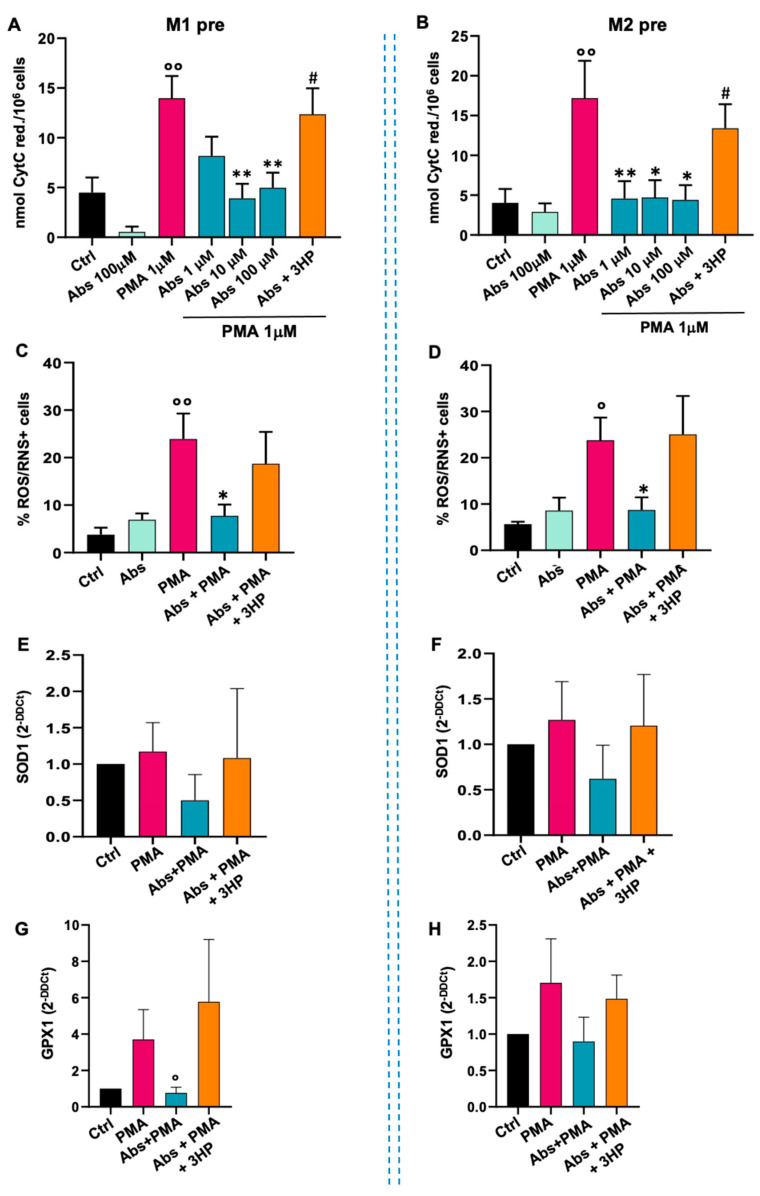
Effects of absinthin on oxidative stress in M1 and M2 pre-macrophages. Monocytes were incubated with absinthin at the indicated concentrations with/without hTAS2R46 antagonist 3HP (10 μM) before driving macrophage differentiation. Fully differentiated macrophages were then stimulated with PMA (1 μM) for 30 min. (**A**) PMA-induced oxidative burst in macrophages M1 and (**B**) in macrophages M2. Data are expressed as nmol of reduced cytochrome C/10^6^ cells and indicated as means ± SEM of 6 independent experiments. (**C**) Cytofluorimetric analysis of ROS/RNS-positive macrophages M1 and (**D**) positive macrophages M2, in the presence or absence of the indicated drugs. Data are expressed as mean ± SEM of 4 independent experiments. (**E**) qPCR analysis of SOD1 expression in macrophages M1 and (**F**) in macrophages M2. (**G**) qPCR analysis of GPX1 expression in macrophages M1 and (**H**) in macrophages M2. Data are expressed as mean ± SEM of 4 independent experiments. Results were analysed using one-way ANOVA with a Kruskal–Wallis test for multiple comparisons. Significance levels: ° *p* < 0.05 and °° *p* < 0.01 vs. Ctrl; * *p* < 0.05 and ** *p* < 0.01 vs. PMA; # *p* < 0.05 vs. Abs 10 μM. Abs, absinthin; Ctrl, control (unstimulated cells); PMA, phorbol 12-mystrate 13-acetate; 3HP, 3-hydroxypelenolide.

Through the experiments conducted so far, we demonstrated that absinthin has a significant impact on the PMA oxidative burst, both in monocytes and in macrophages, in acute treatment. Given the immunomodulatory effect of absinthin on the M0 phenotype, we therefore wondered what happens in M1 and M2 macrophages derived from monocytes treated with absinthin before the induction of differentiation. We therefore treated monocytes for 1 h with absinthin at different concentrations and then differentiated them against M1 (GM-CSF, IFNγ and LPS) or M2 (M-CSF, IL4, IL10 and IL13) phenotypes for 6 days. Once differentiated, macrophages (referred to from now as pre-macrophages) were analysed at the resting state or after stimulation with PMA 1 μM for 30 min. As expected, PMA induced a strong response in control pre-macrophages M1 (Figure 6A) and M2 (Figure 6B). Interestingly, M1 and M2 macrophages derived from absinthin-treated monocytes were already resistant to the PMA-induced burst at the lowest concentration of absinthin (Figure 6A,B). The effect of absinthin on ROS/RNS production was even more marked, with a significant inhibition of the PMA-induced increase in both the pre M1 (Figure 6C) and pre M2 (Figure 6D) cells. 

To evaluate the possibility that absinthin drives monocytes’ differentiation towards a macrophage phenotype more resistant to oxidative burst, we used qPCR to analyse the expression of SOD1 and GPX1 in M1 and M2 macrophages derived from absinthin-pretreated monocytes. Surprisingly, the gene expression of SOD1 (Figure 6E,F) and GPX1 (Figure 6G,H) in both macrophage populations had values comparable to the control despite the acute treatment with PMA and the treatment with absinthin that took place 6 days before the analysis. 

### 2.4. Absinthin Protects Cells from DNA Damage

**Figure 7:**
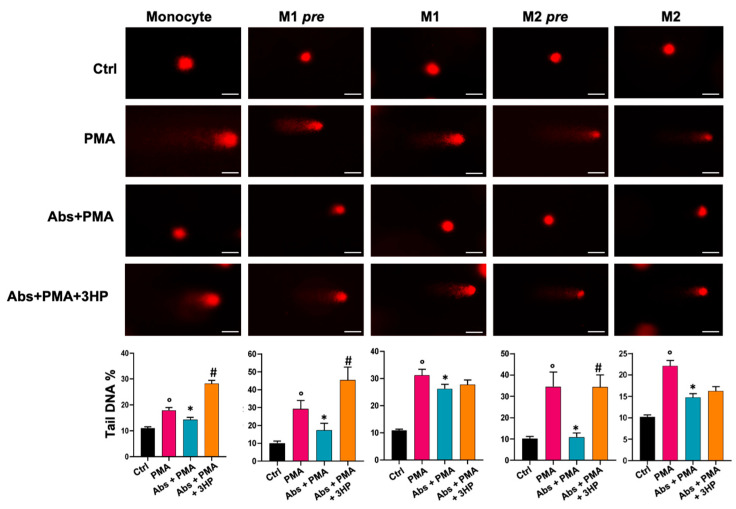
Absinthin protects monocytes and macrophages from PMA-induced DNA damage. Monocytes, fully differentiated macrophages, and macrophages derived from treated monocytes (M1 and M2 pre) were pre-incubated for 1 h with Abs (10 μM) with/without 3HP (10 μM) and then stimulated with PMA (1 μM) for 30 min. Tail DNA percentage was used as a parameter to indicate DNA damage. Data are expressed as the mean ± SEM of at least 50 single cells analysed in three independent experiments. Results were analysed using one-way ANOVA with a Kruskal–Wallis test for multiple comparisons. Significance levels: ° *p* < 0.05 vs. Ctrl; * *p* < 0.05 vs. PMA; # *p* < 0.05 vs. Abs (10 μM). Magnification: 100×. Abs, absinthin; Ctrl, control (unstimulated cells); PMA, phorbol 12-mystrate 13-acetate; 3HP, 3-hydroxypelenolide. Scale bar: 25 μm.

The discovery of absinthin’s ability to inhibit the oxidative burst induced by PMA prompted us to investigate whether absinthin also protected DNA. We therefore prestimulated monocytes with 10 μM absinthin for 1 h, followed by 30 min with 1 μM PMA, and then analysed them for DNA damage using a comet assay or differentiated them against M1 and M2 macrophages (pre M1 and M2). Fully differentiated macrophages derived from untreated monocytes were treated in the same way. As shown in Figure 7, PMA induced significant DNA damage in monocytes and macrophages, which is evident in the representative images of the assay and through the quantification of the percentage of tail DNA reported in the graphs. Absinthin pretreatment protected DNA from PMA-induced damage, especially in monocytes and pre-macrophages, i.e., in those differentiated from monocytes pretreated with absinthin and then induced to differentiation toward macrophages. In fact, in the pre M1 and M2 macrophages, absinthin was more efficient in protecting the DNA from PMA burst, as the percentage of the tail was similar to the control.

The fully differentiated M1 macrophage group treated with absinthin showed significant DNA protection, but this was weaker than that observed in the M2 population (Figure 7).

### 2.5. The Effect of Absinthin on ROS/RNS Correlates with DNA Repair

**Figure 8:**
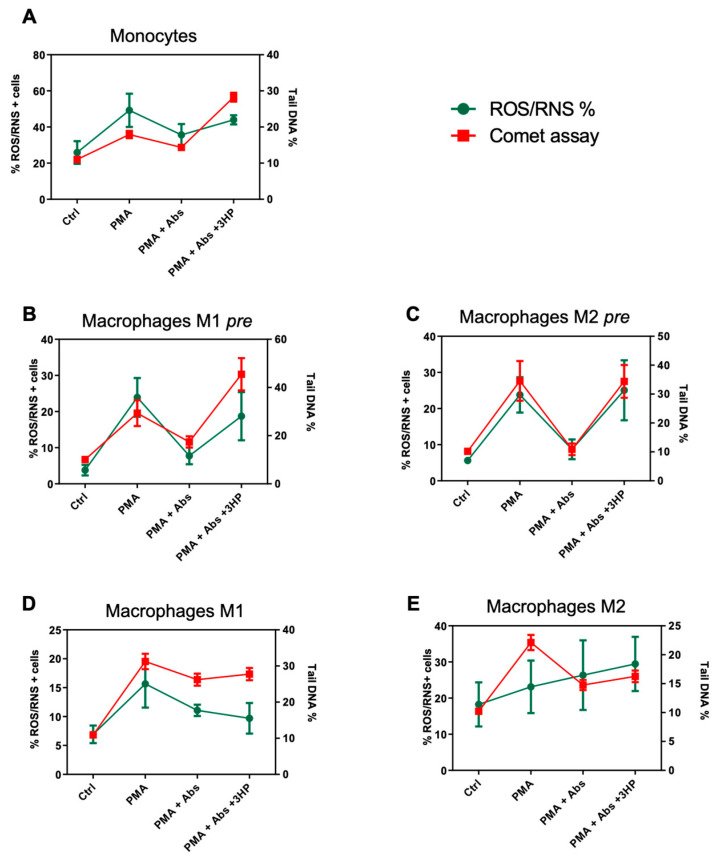
The correlation between the levels of ROS/RNS and oxidative damage to the DNA in (**A**) monocytes, (**B**) M1 macrophages, and (**C**) M2 pre (derived from monocytes treated with absinthin and then differentiated toward macrophages), and (**D**) M1 macrophages and (**E**) M2 treated with absinthin and PMA at the end of differentiation. Red line represents the DNA damage as a percentage of the DNA tail; green line represents the percentage of ROS/RNS probe-positive cells. Ctrl, control (untreated cells); Abs, absinthin; 3HP, 3-hydroxypelenolide.

Based on the findings of oxidative stress and that the ROS bursts of responsive cells result in DNA damage, we related the two processes to the antioxidant and protective effect of absinthin to support our thesis. As shown in Figure 8, the lines of ROS/RNS production and of PMA-induced DNA damage are superimposable: when the PMA-induced oxidative burst was elevated, the damage to DNA was also increased. In monocytes (Figure 8A), in M1 (Figure 8B) and M2 (Figure 8C) pre-macrophages, the stains for ROS/RNS and for tail DNA damage were overlaid both at rest and after treatment with PMA; however, above all, the two curves coincided in the presence of absinthin, which reduces both effects, thus demonstrating the consequentiality of the events. 

The correlation of fully differentiated macrophages (Figure 8D,E) was less marked, especially in the M2 macrophages (Figure 8E), but the responsiveness to the PMA and to absinthin was, in any case, exhaustive.

## 3. Discussion

Absinthin is a specific agonist of the bitter taste receptor TAS2R46 [31] that differs from other bitter agonists, such as strychnine and denatonium, by its characteristic chemical structure, as it belongs to the sesquiterpene lactone family, and its antioxidant and anti-inflammatory properties have been demonstrated in vivo and ex vivo in murine cell models [32,33]. Moreover, we previously demonstrated its antioxidant ability in a human bronchial epithelial cell line [9], confirming a protective role of the bitter taste receptor in the lung. Human monocytes/macrophages express diverse subtypes of bitter taste receptor, including TAS2R46, for which a role has been recognized in the innate immune response [20]. In our study, ex vivo experiments were performed to evaluate the possible antioxidant effects of absinthin in human monocytes/macrophages, using two different experimental approaches: we evaluated the ability of absinthin to modulate the responsiveness of the monocytes/macrophages to PMA-induced oxidative stress and to protect the DNA from oxidative damage, as well as its ability to modulate the differentiation of the monocytes towards a macrophagic phenotype more resistant to oxidative stress. 

First, we have shown that Tas2R46 is the most expressed subtype among the four evaluated (i.e., compared to Tas2r10, 14 and 47), and in turn, it is more expressed in monocytes than in macrophages, with an expression superimposed between that in the M1 and M2 populations. Moreover, absinthin does not immunomodulate monocytes’ phenotype but is able to prime monocytes’ differentiation towards an alternative macrophage population, as M0 macrophages derived from absinthin-treated monocytes present a higher expression of CD163/206 and a decrease in CD80/86. Under our experimental conditions, absinthin could reduce the oxidative burst induced by PMA in monocytes and M1 and M2 macrophages, and, in parallel, prevent the PMA-induced changes in SOD1 and GPX1 expression, restoring it to baseline. Superoxide dismutases (SODs) catalyse the reaction of superoxide into O_2_ and H_2_O_2_ at the site of superoxide generation, while glutathione peroxidase 1 reduces cytoplasmic H_2_O_2_ [29]. Hence, our demonstration that enzyme levels return to the basal expression in the presence of absinthin is confirmation that the bitter ligand hampers the responsiveness of cells to the PMA-induced oxidative burst, therefore resulting in less of the superoxide anion and less hydrogen peroxide being metabolized by SOD1 and GPX1, respectively.

To further understand the antioxidant potential of absinthin, we next evaluated its ability to prime resting monocytes towards macrophage differentiation in response to a cocktail of specific cytokines. Additionally, in this case, the pretreatment of monocytes with absinthin significantly reduced the PMA-induced burst in the differentiated M1 and M2 macrophages, even 6 days after treatment. This suggests that pretreating monocytes with absinthin makes the resulting macrophages resistant to PMA stimulation, therefore preventing the induction of the pro-oxidant machinery and, meanwhile, supporting differentiation towards the M2 population. The predisposition of absinthin-treated monocytes to acquire an antioxidant phenotype was also demonstrated in this experimental condition by the decrease in SOD1 and PGX1 gene expression in monocytes, as well as in both macrophage populations. Indeed, TAS2R46 activation by absinthin suppressed the PMA-induction of the inflammatory genes by interfering with the oxidative pathways in activated monocytes/macrophages. These data raise important questions regarding the role of bitter receptors in monocytes and macrophages. It has been demonstrated that monocytes are DNA-repair-defective and more susceptible to oxidative damage because they lack certain DNA-protective enzymes [34,35], but as we have shown, they express higher levels of TAS2R46 than the macrophages. Thereafter, the transition from monocytes to macrophages involves an increase in the expression of DNA repair enzymes, and therefore, mature differentiated cells acquire greater resistance to ROS-induced DNA damage [36]. We can hypothesize that monocytes partially compensate for the lack of repair enzymes with the greater expression of the bitter taste receptor, which plays a protective role in the body. In fact, following experimental ROS exposure, absinthin significantly hampered DNA damage in monocytes with equal efficacy to that in macrophages despite the lower expression of TAS2R46 in the latter, although this was probably supported by the presence of the whole enzymatic pattern for DNA protection. However, we cannot ignore the presence of the other receptor subtypes to which absinthin can bind, but since the affinity towards subtype 46 is higher and the antagonist 3-hydroxypelenolide reverts its effects, all this leads us to hypothesize that the antioxidant and protective effects of absinthin are mostly TAS2R46-dependent. What role does TAS2R46 play in vivo after pathogen invasion? Acyl-homoserine lactones (AHLs) are quorum-sensing molecules secreted by Gram-negative bacteria that belong to the lactones, a group of chemicals that bind to both TAS2R46 [31] and TAS2R38 [19]. Lactones are demonstrated to modulate host immune cells, such as neutrophils [37], macrophages [38], and T cells [39], and to trigger the airway epithelium with a consequent inflammatory response by TAS2Rs activation [40]. We can therefore speculate that, in the presence of an infection, there will be an increase in activated lymphocytes, neutrophils and monocytes/macrophages with a consequent increase in ROS levels in activated neutrophils monocytes/macrophages to counteract the pathogen invasion. Meanwhile, in lactones secreted by bacteria, on the one hand, they regulate the expression of the virulence genes of the microorganisms, and on the other, they bind to the bitter taste receptors, triggering a protection mechanism in the host immune cell system. Hence, the effects induced by AHLs could be assumed to be TAS2R-mediated.

## 4. Materials and Methods

### 4.1. Monocytes’ Isolation and Differentiation

Seven anonymous human buffy coats were provided by the Transfusion Service of Ospedale Maggiore della Carità (Novara, Italy) after authorization from the local Ethics Committee (authorization document 88/17). Monocytes were isolated using the standard technique of dextran sedimentation and Histopaque (density = 1.077 g cm^−3^, Sigma-Aldrich, St. Louis, MO, USA) gradient centrifugation (400× *g*, 30 min, room temperature) and recovered through thin suction at the interface, as described previously [9]. Purified monocyte populations were obtained by adhesion (90 min, 37 °C, 5% CO_2_) in serum-free RPMI 1640 medium (Sigma-Aldrich) supplemented with 2 mM glutamine and antibiotics. Monocytes were used as is or differentiated into monocyte-derived macrophages (MDMs). The protocols for obtaining the MDM populations were as follows: for M0, cells were cultured in 20% FBS-enriched medium for 7 days; for M1, cells were cultured in 10% FBS-enriched medium with hrGM-CSF (50 ng mL^−1^) for 5 days, and then hrIFN-γ (20 ng mL^−1^) and LPS (50 ng mL^−1^) were added for an additional 24 h; and for M2, monocytes were cultured in 10% FBS-enriched medium added to hrM-CSF (50 ng mL^−1^) for 5 days, and then hrIL-4, hrIL-13, and hrIL-10 (20 ng mL^−1^, all Immunotools) were added for an additional 24 h [41]. MDMs were obtained from both non-treated monocytes and cells stimulated with 10 μM absinthin. It is worth mentioning that each MDM population is not completely pure, but a minimal percentage of non-polarized cells of each specific phenotype can be present in each population.

Monocytes were treated and analysed as such or treated as requested for each specific assay, then washed and left to differentiate into macrophages (hereafter referred to as pre-macrophages), and then analysed. Fully differentiated M1 and M2 macrophages (hereafter referred to as *post* macrophages) were treated and analysed.

### 4.2. Flow Cytometry Analysis

Surface marker expression was measured through multiparametric analysis using flow cytometry (Attune NxT, Life Technologies, Carlsbad, CA, USA). The following antibody panels were used: APC anti-CD14, FITC anti-CD16, PE anti-CD86, FITC anti-CD80, PE anti-CD163, and PerCp anti-CD206. The monocyte and macrophage populations were defined as CD14+ cells. Data are therefore expressed as the numbers of CD86+, CD80+, CD163+, or CD206+ cells over the number of CD14+ cells. CD80 and CD86 are M1-like markers, while CD163 and CD206 are M2-like markers. A comparison between treated and non-treated cells was performed, and the data are expressed as percentages of positive events.

### 4.3. Analysis of Reactive Oxygen and Nitrogen Species (ROS/RNS) Production

M1 and M2 monocytes and macrophages (2.5 × 10^5^ cells/well in a 24-well plate) were treated for 1 h with absinthin alone (0.1 μM, 1 μM, and 10 μM) or in the presence of 1 µM phorbol 12-myristate 13-acetate (PMA; Sigma-Aldrich) for 30 min. Superoxide anion production was then evaluated using a superoxide dismutase (SOD)-sensitive cytochrome C reduction assay and is expressed as nmoles of reduced cytochrome C/10^6^ cells/30 min, using an extinction coefficient of 21.1 mM. 

Moreover, we evaluated the percentage of cells producing ROS/RNS using the Cellular ROS/Superoxide Detection Assay Kit (Abcam ab139476, Cambridge, UK) according to the manufacturer’s instructions. Data were collected and analysed through flow cytometry (Attune NxT Flow Cytometer—Thermo Fisher Scientific, Waltham, MA, USA) and are expressed as the percentages of cells positive in ROS/RNS probe staining.

### 4.4. Quantitative Real Time-PCR

For a gene expression analysis of monocytes and macrophages, the total RNA was isolated using Trizol reagent (Società Italiana Chimici, Roma, Italy), and cDNA synthesis was performed using a high-capacity SensiFAST™ cDNA Synthesis Kit (Bioline) according to the manufacturer’s instructions. 

For TAS2R10, 14, 30, and 46, superoxide dismutase (SOD) 1, and glutathione peroxidase (GPX) 1, a two-step cycling real-time PCR was carried out in a volume of 10 μL per well in a 96-well optical reaction plate (Biorad, Milan, Italy) containing Sensifast No-ROX kit (Bioline, London, UK) 1×, forward and reverse primers at 400 nM, and 1 μL of cDNA template. The primers used were TAS2R10: forward 5′-GACTTGTAAACTGCATTGACTGTG-3′, reverse 5′-GCTGGTGGCAAACCACATAC-3′; TAS2R14: forward 5′- GCTTTGGCAATCTCTCGAATTAG-3′, reverse 5′-TGTCCAGATATTAGTAAGCATTCTG-3′; TAS2R30: forward 5′-GTTATTACTACATTGGTATGCAACTC-3′, reverse 5′-GAGGCTAGTAGCAAGCCAGCT-3′; TAS2R46: forward 5′-GAGTTGAATCCAGCTTTTAACAG-3′, reverse 5′-GGCAATCTTGAGCAAATAAAATATGC-3′; SOD1: forward 5′-GTGGGCCAAAGGATGAAGAGA-3′, reverse 5′-ATAGACACATCGGCCACACC-3′; GPX1: forward 5′-CCAGTTTGGGCATCAGGAGAA-3′, reverse 5′-CGAAGAGCATGAAGTTGGGCT-3′; and GAPDH (internal control): forward 5′- AACGTGTCAGTGGTGGACCTG-3′, reverse 5′- AGTGGGTGTCGCTGTTGAAGT-3′.

Moreover, TAS2R46 expression was confirmed through a TaqMan expression assay. This assay was carried out in a volume of 10 μL per well in a 96-well optical reaction plate (Steroglass, San Martino, Italy) containing 0.5 μL of TaqMan Expression Assay (TAS2R46, Hs00853124_s1, Invitrogen, Waltham, MA, USA), 2.5 μL of RNase-free water, 5 μL of TaqMan Universal PCR MasterMix (2×) (without AmpErase UNG; Applied Biosystem, Waltham, MA, USA), and 1 μL of cDNA template, as described [11]. The plate was run on the 7000 ABI Prism system (Applied Biosystems). To compensate for variations in cDNA concentrations and PCR efficiency between tubes, an endogenous control gene (β-glucuronidase) was included for each sample and used for normalization. The data are expressed as 2^−ΔCt^. The relative quantification was performed using the DCT method. 

### 4.5. Immunofluorescence Assay

For immunofluorescence staining, 2 × 10^5^ cells were plated on a 12 mm diameter glass dish, fixed in 4% PAF for 10 min, and then incubated with the blocking buffer (3% BSA, 0.1% Triton X-100 in PBS) for 1 h at room temperature (RT). Then, cells were incubated for 2 h at RT with a polyclonal rabbit anti-human hTAS2R46 antibody (Thermo Fisher). Then, cells were incubated for 45 min at RT in the dark with the secondary antibody goat anti-rabbit AlexaFluor 488 (Thermo Fisher). DAPI (Sigma-Aldrich) was added to the secondary antibody solution for nuclei staining. Images were acquired using Leica LAS-X software (Version 3.3.0.16799).

### 4.6. Single-Cell Gel Electrophoresis 

To evaluate DNA damage within single cells, we performed a comet assay following Clementi et al.’s protocol [42]. Briefly, cells were cultured in a 12-well plate and treated for 1 h with 10 μM absinthin followed (or not) by PMA (10^−6^ M) for 30 min. Cells were mechanically detached, centrifuged, resuspended in 1 mL of cold PBS, and incubated on ice. Then, 250 μL of cell suspension was mixed with 1 mL of melted low-melting agarose (Fisher Molecular Biology, Rome, Italy), transferred onto agarose-coated microscope slides, and incubated in lysis buffer for 1 h at 4 °C and then with the electrophoresis buffer for 30 min at 4 °C. Electrophoresis was performed using a Comet Assay Tank (Cleaver Scientific, Rugby, UK) and run for 30 min at 21 V and 400 mA. Samples were washed and stained with 10 μg/mL propidium iodide (PI) (Immunological Science, Rome, Italy) for 20 min at RT in the dark. After the last wash, pictures were taken using a fluorescence microscope (DS5500B, Leica, Wetzlar, Germany), and the tail DNA percentage was quantified using the automated CometScore 2.0 software (TrikTek, Berlin, Germany).

### 4.7. Statistical Analysis

Statistical analysis among different cell treatments was performed using Student’s paired *t*-test, or a one-way repeated measures ANOVA with a Kruskal–Wallis multiple comparisons test if more than two treatment groups were compared. The data are expressed as the mean ± SEM of ‘n’ independent experiments performed in triplicate, as detailed in the figure legends, and were considered significant at * *p* < 0.05, ** *p* < 0.01, and *** *p* < 0.001.

## 5. Conclusions

In summary, our data confirm the important involvement of subtype 46 of the large family of bitter taste receptors in our sentinel system by protecting monocytes and macrophages from damage to DNA induced by ROS. In particular, our data support the hypothesis that TAS2R46 can compensate for the lack of DNA repair enzymes in monocytes, therefore limiting their vulnerability to oxidative stress. These results add a piece to the complicated puzzle regarding the function and regulatory mechanisms of bitter taste receptors, which differ greatly depending on the ectopic site of expression.

## Data Availability

The data presented in this study are available in the manuscript.
